# Modeling treatment of diabetic wounds with oxygen therapy and senolytic drug

**DOI:** 10.1038/s41598-025-02852-9

**Published:** 2025-05-23

**Authors:** Nourridine Siewe, Avner Friedman

**Affiliations:** 1 School of Mathematics and Statistics, Rochester Institute of Technology, Rochester, NY USA; 2 Department of Mathematics, The Ohio State University, Columbus, OH USA

**Keywords:** Computational models, Applied mathematics, Statistics, Differential equations

## Abstract

Diabetic wounds are common in patients with type 2 diabetes; they are ischemic and inflammatory, and difficult to heal without intervention. Hyperbaric oxygen therapy (HBOT) is a standard treatment, but its effectiveness is limited to a subset of the aging population. Senescent fibroblasts, a hallmark of aging, impair wound healing, and senolytic drugs, like quercetin (*Q*), which target senescent cells, may improve healing. In this study, we developed a mathematical model that defines biological aging through two parameters, $\eta$ and $A_0$, that decline with age. These parameters reflect the biological age of an individual, where $\eta$ represents fibroblast proliferation and $A_0$ represents the production of the angiogenetic protein VEGF. Our model predicts that treatment with only HBOT achieves wound closure, within normal expectable time, for patients with a limited subset pairs of $(\eta ,A_0)$, and this subset is increased to a larger subset by combining *Q* with HBOT. The two subsets of $(\eta ,A_0)$ are determined explicitly by simulations of the model. To make these results applicable in clinical setting, one will have to relate the aging parameters $\eta$ and $A_0$ to tangible marks of biological-aging factors.

## Introduction

The process of wound healing is divided into four overlapping stages: homeostasis, inflammatory, proliferation, and remodeling. In homeostasis, immediately after injury, platelets from damaged capillaries in the wound bed release PDGF, which stimulates resident fibroblasts^1^, who are then attracted to the wound and begin to secrete PDGF^2^. Actually, neutrophils are one of the first cells that are recruited to the site of the wound. Their primary role is to prevent infection by attacking any microbe attempting to invade the body through the open skin wound^3^. For simplicity, we do not include in our model neutrophils and this very early phase of inflammation. In the inflammatory phase, blood monocytes are attracted to the wound microenvironment, where they differentiate into inflammatory macrophages $M_1$^4^ that produce inflammatory cytokines, in particular TNF-$\alpha$^5^. Fibroblasts secrete TGF-$\beta$^6^, which induces polarization from M1 to anti-inflammatory M2 macrophages^7^, and M2 macrophages secrete TGF-$\beta$^5^. TNF-$\alpha$ induces polarization from M2 to M1 macrophages^8^. In the proliferation phase, M2 macrophages and fibroblasts secrete VEGF^4,9^, and VEGF promotes angiogenesis by stimulating proliferation of endothelial cells and the blood capillary system, thereby increasing the supply of oxygen to the cells in wound microenvironment^10^ and enabling growth of tissue into the wound. Collagen deposition by fibroblasts, promoted by TGF-$\beta$^11^, enables wound closure and end of the actual healing of the wound, except for wound remodeling. The fourth phase of wound remodeling and scar formation may take many months. Here we focus on the first three phases and on wound closure.

In normal dermal wound healing, the inflammatory phase takes at most one or two weeks, and wound closure takes weeks. Such wounds are called acute wounds. Wounds that do not heal in normal expectable time are called chronic wounds. Chronic wounds are consistently inflamed and may not heal without intervention. Chronic wounds include diabetic foot ulcer, ischemic wounds (arterial insufficiency), and pressure ulcer. Chronic wounds are more common in older rather than younger individuals^12,13^. Reviews of chronic wounds and potential therapies in adults are given in^12–15^.

Cellular senescence is a permanent arrest of normal cell cycle, while maintaining cell viability. Senescent cells secrete senescence-associated secretory phenotype (SASP) which include variety of proteins. Cellular senescence is the primary hallmark of aging, but tissue disruption associated with cutaneous wounds also gives rise to senescent cells^16^. SASP of senesent fibroblasts secrete VEGF^17^ and IL-6^18,19^.

A review of cellular senescence in skin aging and age related pathologies, including dermal wounds, is given in^20^. The total number of fibroblasts is reduced by 35% in aged skin (>80 years) while the number of senescent fibroblasts is increased significantly with age^21,22^. Senescent fibroblasts in aging negatively affect dermal wound healing, and may lead to chronic wounds^23^. Senescent fibroblasts and macrophaes, exacerbate inflammation by secreting IL-6^14,19^, which, in chronic wounds, enhance M2$\rightarrow$M1 polarization^18,24^. The production of VEGF by macrophages and fibroblasts in wound healing is reduced in aging^25^ by cellular senescence^26^, resulting in impaired angiogenesis^25,26^.

In this paper we consider diabetic wounds: wounds that are more common in patients with diabetes type 2. Such wounds are consistently inflammatory. Indeed the rate of $M_1/M_2$ correlates with the degree of insulin resistance^27^. This means that the polarizations M1$\rightleftarrows$M2 are weighted toward M1, or that the normal transition from M1 to M2 is partially blocked.

Diabetic dermal wounds (e.g., diabetic foot ulcer) are ischemic, while the adaptive response to hypoxia is impaired in diabetes due to hyperglycemia^28^. Diabetic wounds are treated by oxygen therapy, either by topical wound therapy^29^, or by hyperbaric oxygen therapy^30–32^ where the patient spends several hours daily in the hyperbaric chamber where the oxygen pressure is three times the pressure in air.

Senolytic drugs (e.g., quercetin, dasatinib, fisetin) are drugs that eliminate senescent cells. Such drugs are currently under study in diseases that are exacerbated in aging patients. In the present paper we consider diabetic wounds treated by combination of hyperbaric oxygen therapy (HBOT) and quercetin (Q). Experimental studies report that quercetin improves wound healing^33^ and wound closure^34^.

In this paper, we develop an age-structured mathematical model for diabetic wounds, represented by a system of partial differential equations. We use this model to evaluate the effectiveness of combining Q with HBOT in improving wound closure in diabetic patients.

We conduct simulations to assess the percentage of wound closure in patients of various ages under treatment with HBOT alone, Q alone, and combination of Q and HBOT.

Here “age” is defined by two parameters, $A_0$ and $\eta$ ($0\le A_0\le 1,\ 0\le \eta \le 1$), which are increasing with chronological age. We determine the set of points $(\eta ,A_0)$ where wound closure in normal expectable time can be achieved by combining Q with HBOT, but not by HBOT alone.

**Fig. 1:**
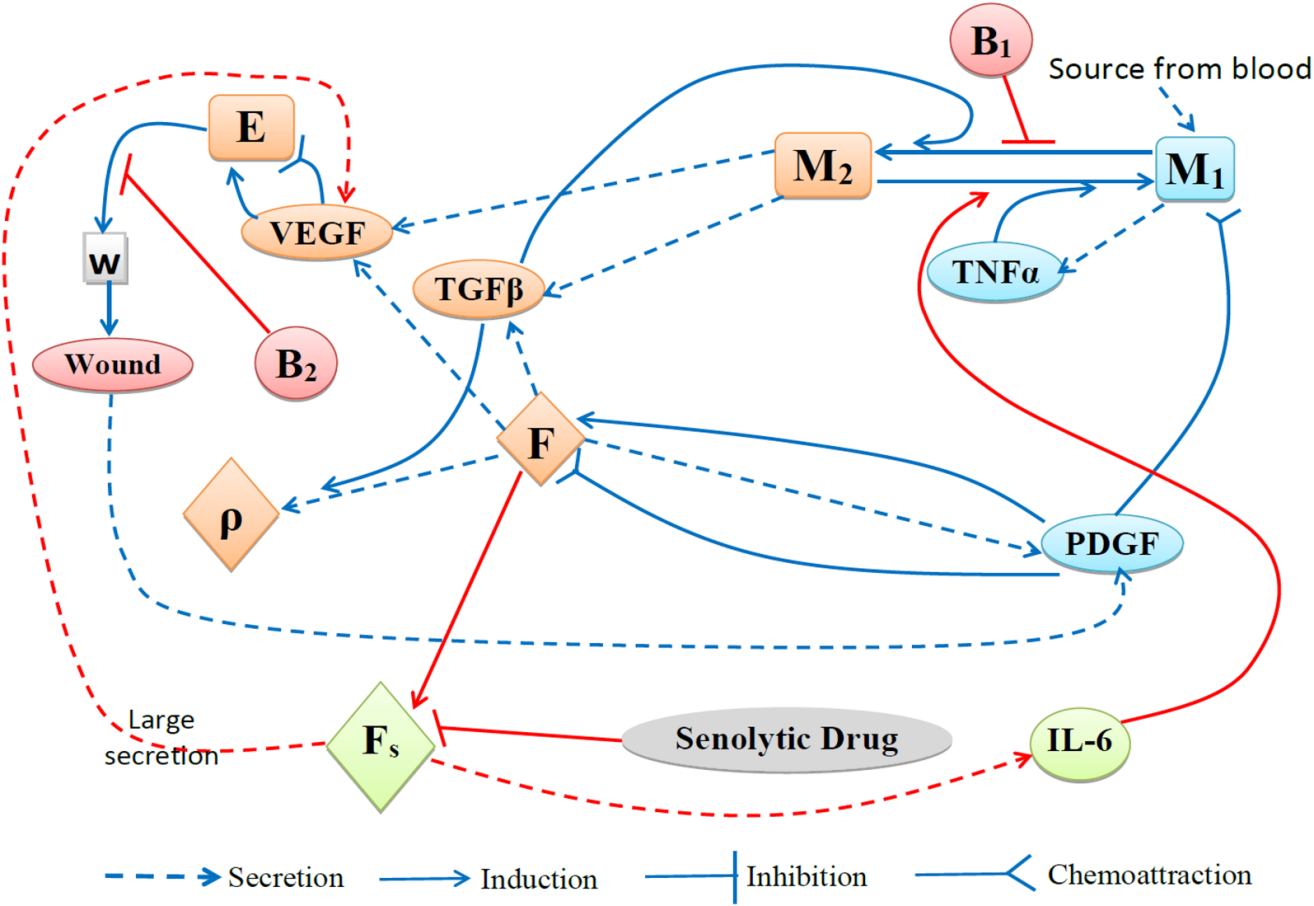
Network of diabetic wound in aging. *w*=oxygen, *E*=endothelial cells, *F*=fibroblast, $F_s$=senescent fibroblast, $\rho$=ECM. $B_1$ and $B_2$ represent blockades in diabetic wounds.

**Fig. 2:**
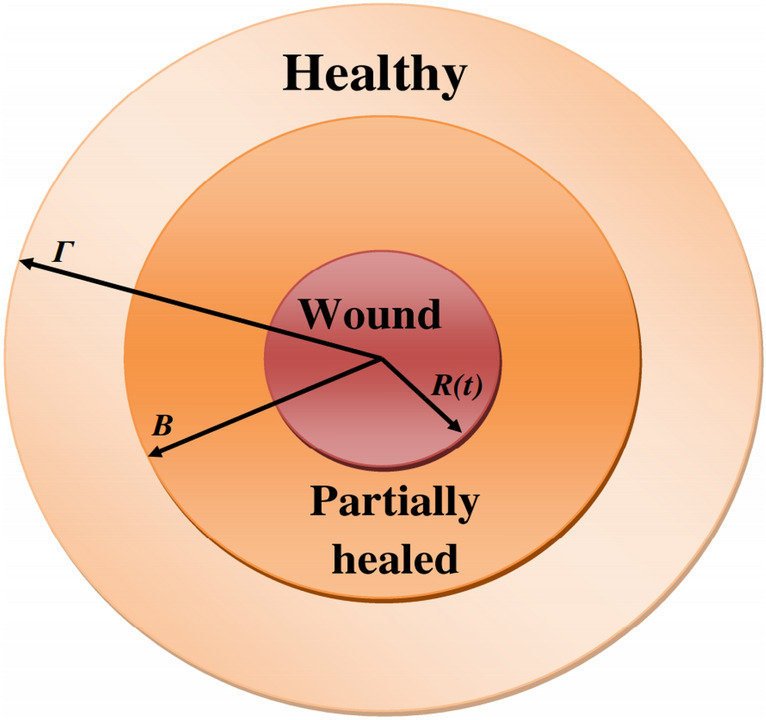
Geometry around the wound. Wound area is $\{0\le r\le R(t)\}$, partially healed area is $\{R(t)\le r\le B\}$, healthy area is $\{B\le r\le \Gamma \}$. This figure was reproduced from^35^.

## Mathematical model

The mathematical model is based on the network shown in Fig. 1, where the blue connections represent the network for acute wound healing, and the red connections represent the network for chronic wound healing. The blue connections in Fig. 1 exclude senescent cells and diabetes-related variables. In this case, we assume the wound heals in normal expectable time without any treatment. On the other hand, the red connections in Fig. 1 incorporate perturbations to the healing process caused by aging (senescent cells, denoted by $F_s$) and diabetes (denoted by $B_1$ and $B_2$), which contribute to the development of chronic wounds. Table 1 lists the variables of the model; densities and concentrations are all in units of g/$\hbox {cm}^3$.

**Table 1:** Variables used in the model. Densities and concentrations are in units of g/$\hbox {cm}^3$.

Variables	Descriptions	Variables	Description
$M_1$	Density of M1 macrophages	$M_2$	Density of M2 macrophages
*E*	Density of endothelial cells	*F*	Density of fibroblasts
$F_s$	Density of senescent fibroblasts	$\rho$	Concentration of ECM
$T_\alpha$	Concentration of TNF-$\alpha$	$T_\beta$	Concentration of TGF-$\beta$
$I_6$	Concentration of IL-6	*P*	Concentration of PDGF
*V*	Concentration of VEGF	*w*	Concentration of oxygen

The wound region is assumed to be a cylinder with an axis normal to the skin and, for simplicity, we consider only the two-dimensional circular cross-section (e.g., the base of the cylinder) as in Fig. 2, with time-varying boundary defined by $r = R(t)$. The partially healed tissue is the shell $R(t) \le r \le B$, while the shell $B \le r \le \Gamma$ represents normal healthy tissue. For simplicity, as in^36^, we ignore the thickness for the wound and assume that the “flat” wound is radially symmetric, depending solely on (*r*, *t*). Throughout the healing process, the extracellular matrix (ECM) and its density $\rho$ experience continuous movement with velocity $v=v(r,t)$, and that $\rho$ is increasing.

To determine the velocity *v*, we adopt the approach outlined in^36^, assuming that the partially healed tissue exhibits viscoelastic properties, which we model as a single-phase upper convected Maxwell fluid with pressure depending on its density: elastic over short timescales and viscous over longer ones. Given the slow dynamics of the healing process, we treat it as quasi-static. Letting $\Psi$ represent the internal isotropic pressure associated with $\rho$ in the partially healed medium, we use the following equation for *v*:

$$\begin{aligned} \frac{1}{r} \frac{\partial }{\partial r} \left( r \frac{\partial v}{\partial r} \right) - \frac{v}{r^2} = \frac{\partial \Psi }{\partial r}, \end{aligned}$$

where $\Psi$ is defined as in^36^ as follows:

1 $$\begin{aligned} \Psi = {\left\{ \begin{array}{ll} 0, & \text {if } \rho \le \rho _1 \\ \gamma \left( \frac{\rho }{\rho _1} - 1 \right) , & \text {if } \rho > \rho _1 \end{array}\right. } \end{aligned}$$

for some positive parameters $\gamma$ and $\rho _1$.

Fibroblasts and M2 macrophages are sensitive to hypoxia due to their dependence on oxidative phosphorylation, whereas M1 macrophages rely on glycolysis for respiration and are not affected by low oxygen levels^37,38^. Accordingly, we assume that the growth rates of fibroblasts *F* and M2 macrophages (but not M1 macrophages) are proportional to:

$$\begin{aligned}G(w) = \frac{w}{w_0 + w},\end{aligned}$$

while the apoptotic death rate for these cells increases in proportion to:

$$\begin{aligned}D(w) = H(w_i - w),\end{aligned}$$

where $w_0$ represents the oxygen concentration in healthy tissue, $w_i$ denotes the oxygen level in extreme hypoxia, and *H*(*s*) is defined as 0 if $s < 0$ and 1 if $s > 0$.

We assume that all cells within the partially healed tissue are moving with the same radial advection velocity *v*, and, in addition, they undergo diffusion. We can then write the dynamics of each species *X* of cells, in the partially healed tissue, in the following form:

2 $$\begin{aligned} \dfrac{\partial X}{\partial t}+\dfrac{1}{r}\dfrac{\partial }{\partial r}(rvX)-D_X\dfrac{1}{r}\dfrac{\partial }{\partial r}\left( r\dfrac{\partial X}{\partial r}\right) =F_X, \end{aligned}$$

where $D_X$ is the diffusion coefficient, and $F_X$ represents the balance of the mass of *X* by the exchanges indicated in Fig. 1. We use the same structural equation for each cytokine *X*, but drop the advection velocity, since it is negligible compared to large diffusion coefficients of cytokines. An expression in $F_X$ of the form $\lambda Z\frac{Y}{K_Y+Y}$ describes a process where species *Y* (e.g., proteins) is absorbed by cells *Z* at rate $\lambda$; $K_Y$ is called the half-saturation of *Y*.

### Equation for ECM ($\rho$)

Fibroblasts produce ECM proteins (e.g. collagen) in a process that is enhanced by TGF-$\beta$^11,35^. We assume that the ECM in the partially healed region is also undergoing advection with the velocity *v*, and write the equation for $\rho$ as follows:

3 $$\begin{aligned} \dfrac{\partial \rho }{\partial t}+\dfrac{1}{r}\dfrac{\partial }{\partial r}(r\rho v)=&\ \lambda _\rho F\left( 1+\lambda _{\rho T_\beta }\dfrac{T_\beta }{K_{T_\beta }+T_\beta }\right) \left( 1-\dfrac{\rho }{\rho _m}\right) -\mu _\rho \rho , \end{aligned}$$

where $\rho _m$, the carrying capacity of ECM, is larger than $\rho _1$ in Eq. (1) and $\mu _\rho$ is the degradation rate of $\rho$.

### Equations for M1 and M2 macrophages

We assume, as in^35^, that there is a constant source of M1 macrophages from the blood to the wound, $A_{M_1}$, and a constant polarization from M1 to M2 macrophages (at rate $\lambda _{M_1M_2}$). According to Fig. 1, PDGF attracts and activates M1 macrophages^4^, and TGF-$\beta$ induces polarization from M1 to M2 macrophages^7^; and IL-6^18,24^ and TNF-$\alpha$^8^ promote polarization of M2 to M1 macrophages. Hence $M_1$ satisfies the following equation:

4 $$\begin{aligned} \dfrac{\partial M_1}{\partial t}&+\dfrac{1}{r}\dfrac{\partial }{\partial r}(rM_1 v)-\dfrac{1}{r}\dfrac{\partial }{\partial r}\left[ r\left( D_{M_1}\dfrac{\partial M_1}{\partial r}\right) \right] =\ A_{M_1}-\dfrac{1}{r}\dfrac{\partial }{\partial r}\left[ r\left( \chi _PM_1\dfrac{\partial P}{\partial r}\right) \right] \nonumber \\&\ +\lambda _{M_1P}M_1^0\dfrac{P}{K_P+P}-\lambda _{M_1M_2T_\beta }M_1\dfrac{T_\beta }{K_{T_\beta }+T_\beta }+\lambda _{M_2M_1T_\alpha }M_2\dfrac{T_\alpha }{K_{T_\alpha }+T_\alpha }\nonumber \\&\ -\dfrac{\lambda _{M_1M_2}}{1+B_1}M_1+\lambda _{M_2M_1I_6}M_2\dfrac{I_6}{K_{I_6}+I_6}-\mu _{M_1}M_1, \end{aligned}$$

where $M_1^0$ is a source of inactive M1 macrophages in the skin, $\mu _{M_1}$ is the death rate, and $\chi _P$ is the chemotactic coefficient of PDGF (*P*).

M2 macrophages are derived from the exchange M1$\rightarrow$M2, their growth rate is proportional to *G*(*w*), and their death rate increases due to a hypoxia. Hence,

5 $$\begin{aligned} \dfrac{\partial M_2}{\partial t}&\ +\dfrac{1}{r}\dfrac{\partial }{\partial r}(rM_2 v)-\dfrac{1}{r}\dfrac{\partial }{\partial r}\left[ r\left( D_{M_2}\dfrac{\partial M_2}{\partial r}\right) \right] = \dfrac{\lambda _{M_1M_2}}{1+B_1}M_1+ \lambda _{M_1M_2T_\beta }G(w)M_1\dfrac{T_\beta }{K_{T_\beta }+T_\beta }\nonumber \\&\ -\lambda _{M_2M_1T_\alpha }M_2\dfrac{T_\alpha }{K_{T_\alpha }+T_\alpha }-\lambda _{M_2M_1I_6}M_2\dfrac{I_6}{K_{I_6}+I_6}-\mu _{M_2}M_2-\mu _{M_2w}D(w)M_2. \end{aligned}$$

### Equation for fibroblasts (*F*) and senescent fibroblasts ($F_s$)

Fibroblasts are attracted to PDGF^2^. As in^35^, we model their proliferation by a logistic growth model, with both proliferation and death rates being dependent on oxygen level. Fibroblasts can become senescent due to a variety of intracellular and extracellular stressors^16^. We assume a constant rate of senescent fibroblasts formation. In order to distinguish between old and young patients, we introduce a parameter $\eta \ge 0$ in that regulates the proliferation of senescent cells. Hence,

6 $$\begin{aligned} \dfrac{\partial F}{\partial t}+\dfrac{1}{r}\dfrac{\partial }{\partial r}(rFv) -\dfrac{1}{r}\dfrac{\partial }{\partial r}\left[ r\left( D_F\dfrac{\partial F}{\partial r}\right) \right] =&\ -\dfrac{1}{r}\dfrac{\partial }{\partial r}\left[ r\left( \chi _PF\dfrac{\partial P}{\partial r}\right) \right] +\lambda _FG(w)F\left( 1-\dfrac{F}{F_m}\right) \nonumber \\&\ -\lambda _{FF_s}(1-\eta )F-\mu _FF-\mu _{Fw}D(w)F \end{aligned}$$

7 $$\begin{aligned} \dfrac{\partial F_s}{\partial t}+\dfrac{1}{r}\dfrac{\partial }{\partial r}(rF_sv) -\dfrac{1}{r}\dfrac{\partial }{\partial r}\left[ r\left( D_{F_s}\dfrac{\partial F_s}{\partial r}\right) \right] =&\ \lambda _{FF_s}(1-\eta )F-\mu _{F_s}F_s, \end{aligned}$$

where, $\lambda _{FF_s}$ and $\eta$ are constants, and $0\le \eta \le 1$. The parameter $\eta$ is decreasing with chronological age^16^.

### Equation for endothelial cells (*E*)

We assume a constant source ($\lambda _E$) of endothelial cells.

VEGF chemoattracts endothelial cells and stimulates their proliferation^10^. Hence *E* satisfies the following equation:

8 $$\begin{aligned} \dfrac{\partial E}{\partial t}+\dfrac{1}{r}\dfrac{\partial }{\partial r}(rEv)&\ - \dfrac{1}{r}\dfrac{\partial }{\partial r}\left[ r\left( D_E\dfrac{\partial E}{\partial r}\right) \right] = -\dfrac{1}{r}\dfrac{\partial }{\partial r}\left[ r\left( \chi _VE\dfrac{\partial V}{\partial r}\right) \right] \nonumber \\&\ +\dfrac{\lambda _E}{1+B_2}\left( 1+\lambda _{EV}\dfrac{V}{K_V+V}\right) -\mu _EE, \end{aligned}$$

where $\chi _V$ is the chemotactic coefficient of *V*.

### Equation for PDGF (*P*)

PDGF is released by damaged platelets from the wounds^39^ and fibroblasts^2^, and PDGF is depleted through chemoattracting *F* and $M_1$. We assume that the source of damaged platelets from the wound is proportional to the radius *R*(*t*) of the wound. Hence PDGF satisfies the following equation:

9 $$\begin{aligned} \dfrac{\partial P}{\partial t} -\dfrac{1}{r}\dfrac{\partial }{\partial r}\left[ r\left( D_P\dfrac{\partial P}{\partial r}\right) \right] =&\ \lambda _PR(t)+\lambda _{FP}F -(\mu _{PF}F+\mu _{PM}M_1)\dfrac{P}{K_P+P}-\mu _PP, \end{aligned}$$

where $\mu _P$ is the degradation rate of *P* and $\lambda _P$ is constant in chronic wounds.

### Equation for TNF-$\alpha$ ($T_\alpha$)

TNF-$\alpha$ is produced by M1 macrophages^5^, hence

10 $$\begin{aligned} \dfrac{\partial T_\alpha }{\partial t} -\dfrac{1}{r}\dfrac{\partial }{\partial r}\left[ r\left( D_{T_\alpha }\dfrac{\partial T_\alpha }{\partial r}\right) \right] =&\ \lambda _{M_1T_\alpha }M_1-\mu _{T_\alpha }T_\alpha , \end{aligned}$$

where $\mu _{T_\alpha }$ is the degradation rate of $T_\alpha$.

### Equation for TGF-$\beta$ ($T_\beta$)

TGF-$\beta$ is produced by fibroblasts and M2 macrophages^6^, so that

11 $$\begin{aligned} \dfrac{\partial T_\beta }{\partial t} -\dfrac{1}{r}\dfrac{\partial }{\partial r}\left[ r\left( D_{T_\beta }\dfrac{\partial T_\beta }{\partial r}\right) \right] =&\ \lambda _{FT_\beta }F+\lambda _{M_2T_\beta }M_2-\mu _{T_\beta }T_\beta . \end{aligned}$$

### Equation for IL-6 ($I_6$)

IL-6 is secreted by senescent fibroblasts^18,19^, so that

12 $$\begin{aligned} \dfrac{\partial I_6}{\partial t} -\dfrac{1}{r}\dfrac{\partial }{\partial r}\left[ r\left( D_{I_6}\dfrac{\partial I_6}{\partial r}\right) \right] =&\ \lambda _{F_sI_6}F_s-\mu _{I_6}I_6. \end{aligned}$$

### Equation for VEGF (*V*)

VEGF is produced by fibroblasts^9^, senescent fibroblasts^17,40,41^ and M2 macrophages^4,9^. The senescent fibroblasts secrete more VEGF than their presenescent counterparts^40,41^. However, in diabetic wounds of old people, VEGF production is decreased by both *F* and $F_s$^25^. VEGF is also depleted when it combines with receptors on endothelial cells. Hence,

13 $$\begin{aligned} \dfrac{\partial V}{\partial t} -\dfrac{1}{r}\dfrac{\partial }{\partial r}\left[ r\left( D_V\dfrac{\partial V}{\partial r}\right) \right] =&\ (\lambda _{FV}F+\lambda _{F_sV}F_s)A(t)+\lambda _{M_2V}M_2-\mu _{EV}E\dfrac{V}{K_V+V}-\mu _VV \end{aligned}$$

where *A*(*t*) is a decreasing function in *t*. We take

14 $$\begin{aligned} A(t)=\dfrac{A_0}{1+t^2/T_w^2},\qquad 0\le A_0\le 1, \end{aligned}$$

where $A_0$ and $T_w$ are a constants. The parameter $A_0$ is decreasing with chronological age.

### Equation for oxygen (*w*)

Hence, *w* satisfies the following equation: We identify the density of blood vessel by *E*. Hence oxygen is increased proportionally to *E*, and is utilized by fibroblasts and M2 macrophages^35^. Hence,

15 $$\begin{aligned} \dfrac{\partial w}{\partial t} -\dfrac{1}{r}\dfrac{\partial }{\partial r}\left[ r\left( D_w\dfrac{\partial w}{\partial r}\right) \right] =&\ \lambda _{Ew}E-c_w(F+M_2)w. \end{aligned}$$

### Boundary conditions

We take the boundary conditions at $r=B$ to be the average serum concentration of the variables in diabetes type 2:

16 $$\begin{aligned}&M_1=M_1^0,\ M_2=M_2^0,\ F=F_0,\ F_s=F_s^0,\ E=E_0,\ w=w_0,\nonumber \\&I_6=I_6^0,\ P=P_0,\ T_\alpha =T_\alpha ^0,\ T_\beta =T_\beta ^0,\ V=V_0, \end{aligned}$$

On the free boundary $r=R(t)$ we prescribe the following conditions:

17 $$\begin{aligned}&D_{M_1}\dfrac{\partial M_1}{\partial r}-\chi _PM_1\dfrac{\partial P}{\partial r}=0,\qquad \dfrac{\partial M_2}{\partial r}=0,\nonumber \\&D_F\dfrac{\partial F}{\partial r}-\chi _PF\dfrac{\partial P}{\partial r}=0,\quad D_E\dfrac{\partial E}{\partial r}-\chi _VE\dfrac{\partial V}{\partial r}=0,\quad -\dfrac{\partial P}{\partial r}=\beta _PR(t),\nonumber \\&\dfrac{\partial X}{\partial r}=0\ \text {for}\ F_s,\ I_6,\ T_\alpha ,\ T_\beta ,\ V,\ w. \end{aligned}$$

Note that in Eq. (17) all species, except *P*, satisfy no-flux condition, while there is an influx of *P* from the wound, proportional to the size of the wound.

The partially healed area has a fixed boundary $r=B$ and a boundary $r=R(t)$ that moves in response to the pressure $\Psi$. Accordingly, we take

18 $$\begin{aligned}&v=0\ \text {at}\ r=B, \end{aligned}$$

19 $$\begin{aligned}&\dfrac{\partial v}{\partial r}=\Psi \ \text {at}\ r=R(t). \end{aligned}$$

We assume that the wound boundary $r=R(t)$ decreases with the velocity *v* of the ECM, that is

20 $$\begin{aligned} \dfrac{dR}{dt}=v(R(t),t); \end{aligned}$$

*v* is expected to have negative values.

### Initial conditions

We take $R(0)=1$ cm, $B=1.5$ cm, and initial conditions for $R(0)\le r\le B$ as follows:

21 $$\begin{aligned}&v(r,0)=0,\ \rho (r,0)=\rho _0,\ P(r,0)=\beta _PR(0)(B-r)+P_0\left( \dfrac{r-R(0)}{B-R(0)}\right) ^2,\nonumber \\&X(r,0)=X_0\left( \dfrac{r-R(0)}{B-R(0)}\right) ^2, \end{aligned}$$

for all other species, where $X_0$ is the steady state value as in (16) and $\rho _0$ is the dry weight of tissue.

Since the level of senescent cells depends on the age of the patient, we take

22 $$\begin{aligned} F_s(0)=F_s^0(1-\eta ), \end{aligned}$$

where $F_s^0$ represents the level of $F_s$ for the oldest patient (with $\eta =0$).

Note that the initial conditions are consistent with the boundary conditions at $r=R(0)$ and $r=B$.

## Simulations and results

All the computations were done using Python 3.7.3. The parameter values of the model equations are estimated in Section 7 and are listed in the Table 4. We introduce the percent of wound closure (PWC) by the formula

23 $$\begin{aligned} \text {PWC}(t)=\dfrac{R(0)-R(t)}{R(0)}\times 100\%. \end{aligned}$$

### Diabetic wounds without senolytics

In the sequel we define “age” by the two parameters, $A_0$ and $\eta$, which are decreasing when the chronological age (*t*) increases. In particular, a person with ($\eta _1,A_{01}$) is older than a person with $(\eta _2,A_{02})$ if $\eta _1<\eta _2$ and $A_{01}<A_{02}$.

**Fig. 3:**
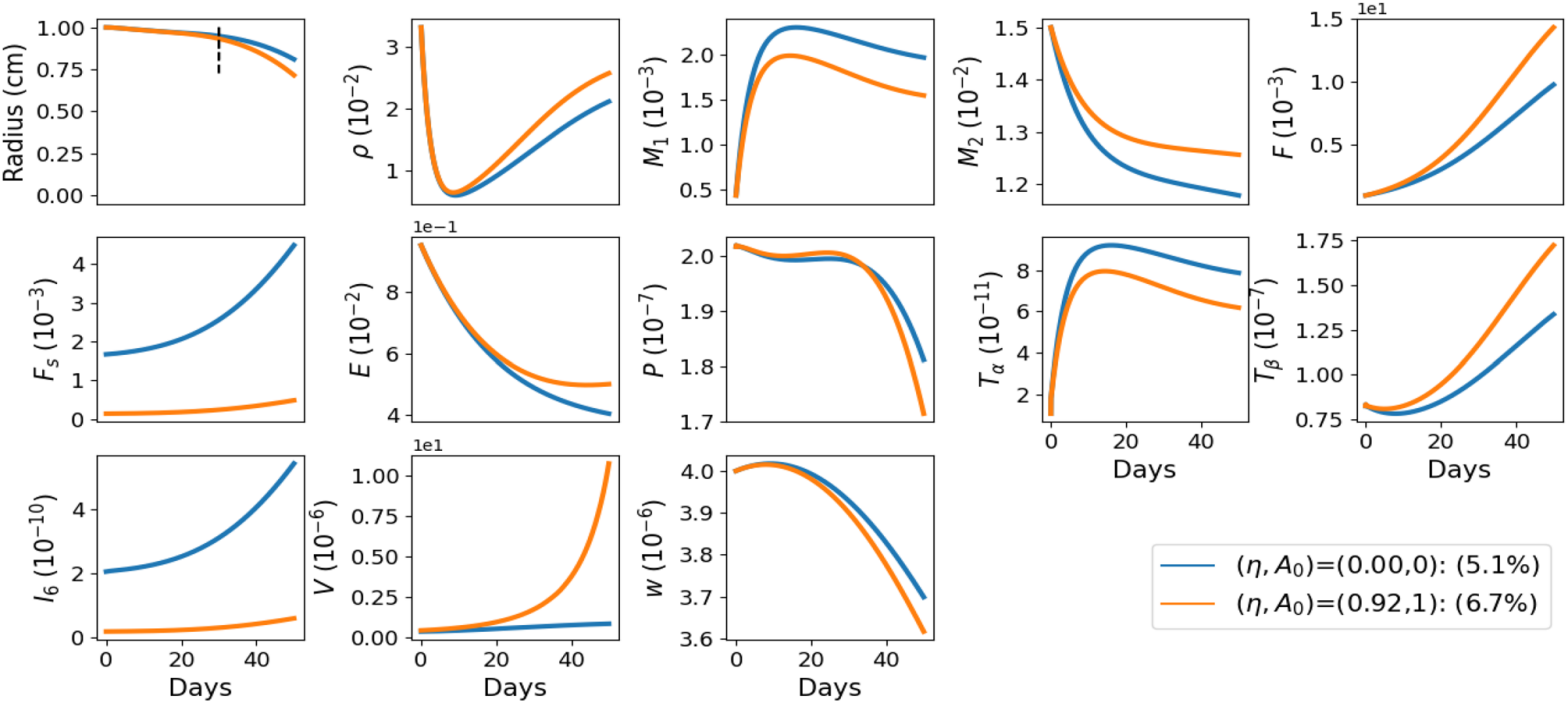
Simulations of all the model variables in oldest patient ($(\eta , A_0)=(0,0)$), and younger patient ($(\eta , A_0)=(0.92,1)$), with no drug. All the variables are in units of g/$\hbox {cm}^3$.

In Fig. 3 we show all the model variables and display the percent of wound closure at day 30 in the no drug case, for the oldest patient and a younger patient. The percentage of wound closure after 30 days is 5.1% in the oldest patient, and 6.7% in the younger patient. In both cases, as expected, the pro-inflammatory $M_1$ increases and tends to stabilize at a high value, while the anti-inflammatory $M_2$ continues to decrease. The oxygen level initially increases but then decreases due to the hypoxia-driven effects of diabetes. The level of senescent fibroblasts, denoted as $F_s$, increases monotonically with age in both the youngest and oldest patients, but the $F_s$ level in the youngest patients remains lower than in the oldest patients. In each case, the level of IL-6 follows the trends observed for $F_s$.

It is interesting to note that whereas VEGF increases to $10\times 10^{-6}$ g/$\hbox {cm}^3$ in young patients, in old patients it increases to only $0.8\times 10^{-6}$ g/$\hbox {cm}^3$. *F* is also smaller in old compared to young patients, and so is $M_2$.

### Simulations with drugs

#### Quercetin

Senolytics, such as quercetin and fisetin, are currently undergoing clinical trials for various diseases like idiopathic pulmonary fibrosis, chronic kidney disease, Alzheimer’s disease, and diabetes^42^. However, there have been no clinical trials to date exploring their effectiveness in wound healing.

Quercetin, a natural supplement, is known to promote wound healing by enhancing the proliferation and migration of fibroblasts^43^ and by eliminating senescent cells^44^. Quercetin also enhances ECM production^13,45^. In combination with dasatinib, a tyrosine kinase inhibitor that triggers apoptosis in senescent cells^46^, quercetin has been shown to significantly reduce the burden of senescent cells across multiple tissues in human studies^46–48^.

Cutaneous wounds were treated with quercetin in mice (*in vivo*)^49,50^ and in humans (*in vitro*)^34^. In all these cases, subjects given the senolytic drugs exhibited faster wound healing compared to the control groups.

We simulate the senolytic drug quercetin, and denote it by *Q*. We accordingly update Eqs. (3), (6) and (7) as follows:

24 $$\begin{aligned} \dfrac{\partial \rho }{\partial t}&\ +\dfrac{1}{r}\dfrac{\partial }{\partial r}(r\rho v)= \lambda _\rho F\left( 1+\lambda _{\rho T_\beta }\dfrac{T_\beta }{K_{T_\beta }+T_\beta }\right) \left( 1-\dfrac{\rho }{\rho _m}\right) (1+\mu _{\rho Q}Q)-\mu _\rho \rho , \end{aligned}$$

25 $$\begin{aligned} \dfrac{\partial F}{\partial t}+\dfrac{1}{r}\dfrac{\partial }{\partial r}(rFv)&\ -\dfrac{1}{r}\dfrac{\partial }{\partial r}\left[ r\left( D_F\dfrac{\partial F}{\partial r}\right) \right] = -\dfrac{1}{r}\dfrac{\partial }{\partial r}\left[ r\left( \chi _PF\dfrac{\partial P}{\partial r}\right) \right] \nonumber \\&\ +\lambda _FG(w)(1+\lambda _{FQ}Q)F\left( 1-\dfrac{F}{F_m}\right) -\lambda _{FF_s}(1-\eta )F-\mu _FF-\mu _{Fw}D(w)F \end{aligned}$$

26 $$\begin{aligned} \dfrac{\partial F_s}{\partial t}+\dfrac{1}{r}\dfrac{\partial }{\partial r}(rF_sv)&\ -\dfrac{1}{r}\dfrac{\partial }{\partial r}\left[ r\left( D_{F_s}\dfrac{\partial F_s}{\partial r}\right) \right] = \lambda _{FF_s}(1-\eta )F-\mu _{F_s}F_s - \mu _{F_sQ}QF_s, \end{aligned}$$

where $\mu _{\rho Q}$, $\lambda _{FQ}$ and $\mu _{F_sQ}$ are constants.

Quercetin is given in pills daily. At 1 g/d it can be given safely (from damage to the liver) for up to 12 weeks. The half-life of quercetin is 15–28h, so by approximation we take it as a constant: $Q=1\ \text {g/d}$ and accordingly estimate the parameters $\lambda _{FQ}$ and $\mu _{F_sQ}$.

#### Oxygen therapy

In hyperbaric oxygen therapy (HBOT) chamber, the oxygen pressure is three times the pressure in air, and the patient remains in the chamber for, typically, 2 hours daily. We represent this treatment in our model by including in Eq. (15) a (constant) source of oxygen, as shown in the following modified equation:

27 $$\begin{aligned} \dfrac{\partial w}{\partial t} -\dfrac{1}{r}\dfrac{\partial }{\partial r}\left[ r\left( D_w\dfrac{\partial w}{\partial r}\right) \right] =&\ \lambda _{Ew}E-c_w(F+M_2)w+\gamma _wh(t), \end{aligned}$$

where

$$\begin{aligned} h(t)={\left\{ \begin{array}{ll} 1, & \ \text {for two hours every day,}\\ 0, & \ \text {otherwise}, \end{array}\right. } \end{aligned}$$

and $\gamma _w>0$ is the dose of oxygen administered between 12:00–14:00 every day. We also change the boundary condition for *w* on $r = B$, increasing $w_0$ by $\int ^t\gamma _w(s)ds$ during the hours between 12:00–14:00.

**Fig. 4:**
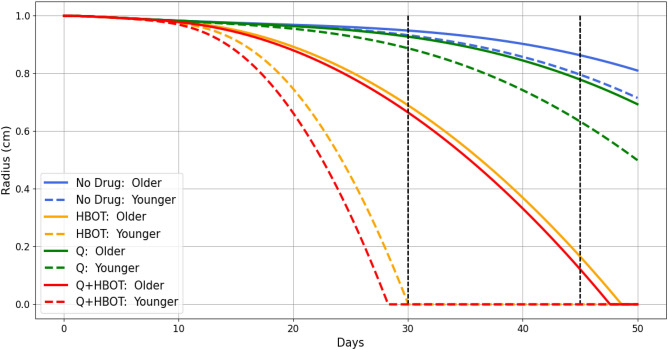
Diabetic wound treatment with HBOT and quercetin: Simulations of the wound radius with various combinations of hyperbaric oxygen therapy (HBOT) and quercetin (Q) for the youngest patient $(\eta ,A_0)=(0.92,1)$ and the oldest patient $(\eta ,A_0)=(0,0)$. The percentages represent the percent of wound closure (PWC) of the respective treatments at day 30; we mark day 30 and day 45.

#### Treatment with HBOT and quercetin

In Fig. 4, we show the profiles of the wound radius (*R*) for 50 days, for two patients of extreme age difference (Younger with $(\eta ,A_0)=(0.92,1)$ and Older with $(\eta ,A_0)=(0,0)$), both with and without drugs HBOT and Q. The profiles for the no-drug case are similar to those in Fig. 3.

Without treatment, diabetic wounds do not close (PWC in 5.1%–6.7%). When using Q alone, there is only a mild improvement in the PWC (between 7.2%–11.2%) compared to the case with no drug.

For younger patients, HBOT treatment does not result in complete wound closure within the expectable 30 days (PWC=99.6%); but when Q is combined with HBOT, complete wound closure is achieved. Older patients, on the other hand, require additional time for complete wound closure.

**Table 2:** Percent of wound closure (PWC) at day 30 in patients of various ages under treatment with hyperbaric oxygen therapy (HBOT) and quercetin (Q). The daily dose of quercetin is 1.0 g, and $\gamma _w=1.2\times 10^{-6}$ g/$\hbox {cm}^3\hbox {d}^{-1}$.

Patients $(\eta ,A_0)$	1 (0.15,0.56)	2 (0.3,0.67)	3 (0.6,0.78)	4 (0.7,0.89)	5 Youngest (0.92,1)
No Drug (%)	5.41	5.67	6.17	6.35	6.71
HBOT (%)	82.29	90.26	96.12	97.48	99.55
Q (%)	7.89	8.53	9.82	10.28	11.24
HBOT+Q (%)	92.56	100	100	100	100

We can use the model to depict cases where complete wound closure can be achieved with Q+HBOT therapy, but not with HBOT alone. Table 2 presents the 30-day PWC for 5 patients, listed in order of decreasing age. In all cases, treatment with only Q does not significantly improve wound closure. Treatment with only HBOT significantly improves PWC but does not fully close the wound. Combining Q with HBOT results in complete wound closure, except for the oldest patient ($(\eta ,A_0)=(0.15,0.56)$).

**Table 3:** Percent of wound closure (PWC) at day 45 in patients of various ages under treatment with hyperbaric oxygen therapy (HBOT) and quercetin (Q). The daily dose of quercetin is 1.0 g, and $\gamma _w=1.2\times 10^{-6}$ g/$\hbox {cm}^3\hbox {d}^{-1}$.

Patients $(\eta ,A_0)$	6 (Oldest) (0,0)	7 (0.001,0.11)	8 (0.003,0.22)	9 (0.004,0.33)	10 (0.03,0.44)
No Drug (%)	13.66	13.72	13.79	13.86	14.11
HBOT (%)	83.41	87.63	95.62	99.47	100
Q (%)	22.09	22.16	22.25	22.33	22.8
HBOT+Q (%)	87.81	92.3	100	100	100

In Table 3, we consider 5 patients older than those in Table 2, and show their PWC after 45 days of treatment. As in Table 2. Treatment with only Q does not result in significant wound closure. Under HBOT alone, the youngest patient (#10, $(\eta , A_0) = (0.03, 0.44)$) achieves complete wound closure. The next two older patients (#8 and #9) achieve wound closure only when Q is combined with HBOT. However, the oldest patients (#6 and #7) do not achieve wound closure with HBOT+Q.

**Fig. 5:**
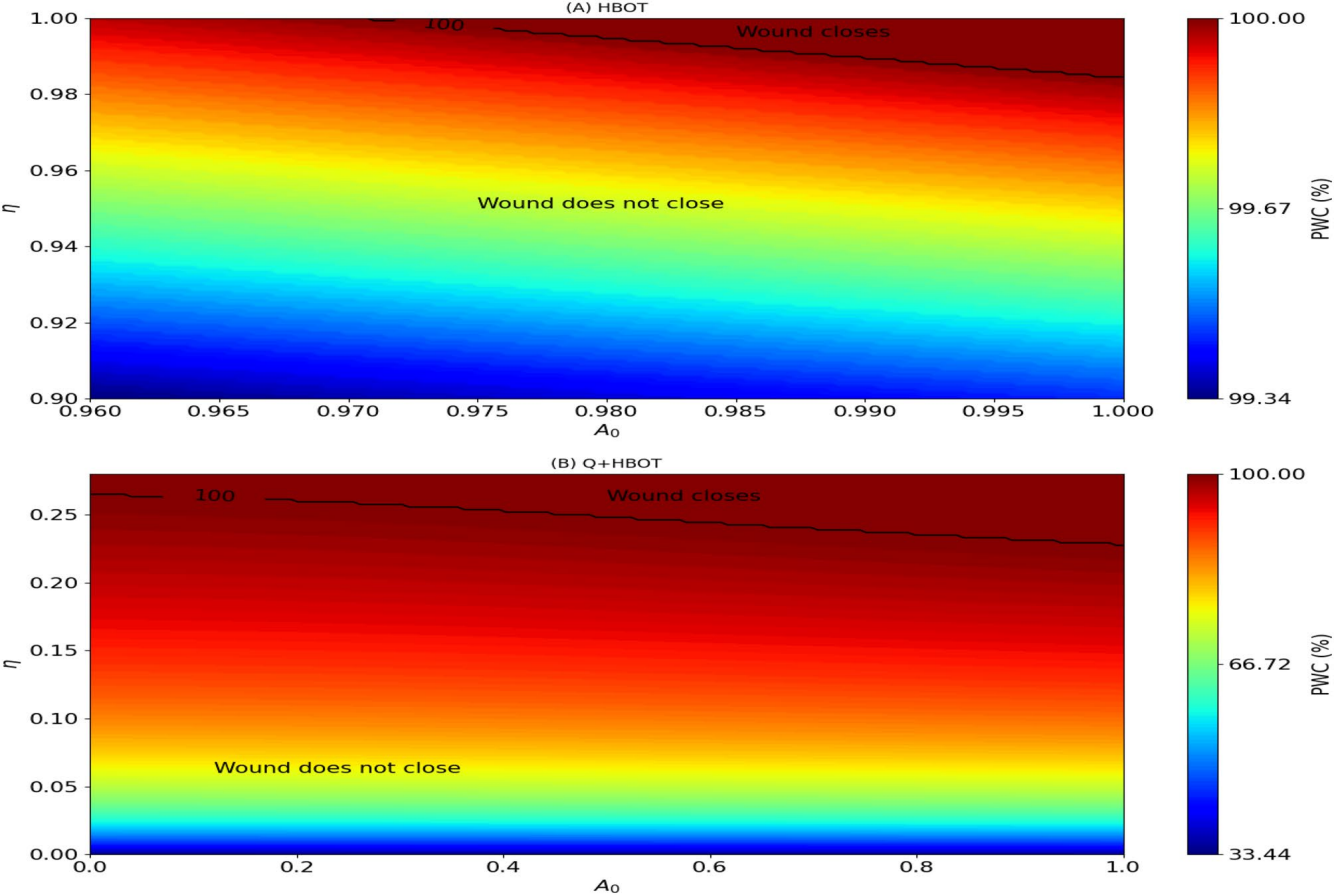
Effect of age $(\eta ,A_0)$ on percent wound closure: Simulations of PWC(30) under HBOT (**A**) and Q+HBOT (**B**) as the pair $(\eta ,A_0)$ vary; note the ranges for $\eta$ and $A_0$ in each plot. The black curves represent the values of $(\eta ,A_0)$ where PWC(30)$=100\%$. The black curves represent the pairs $(\eta ,A_0)$ where PWC(30)=100%.

**Fig. 6:**
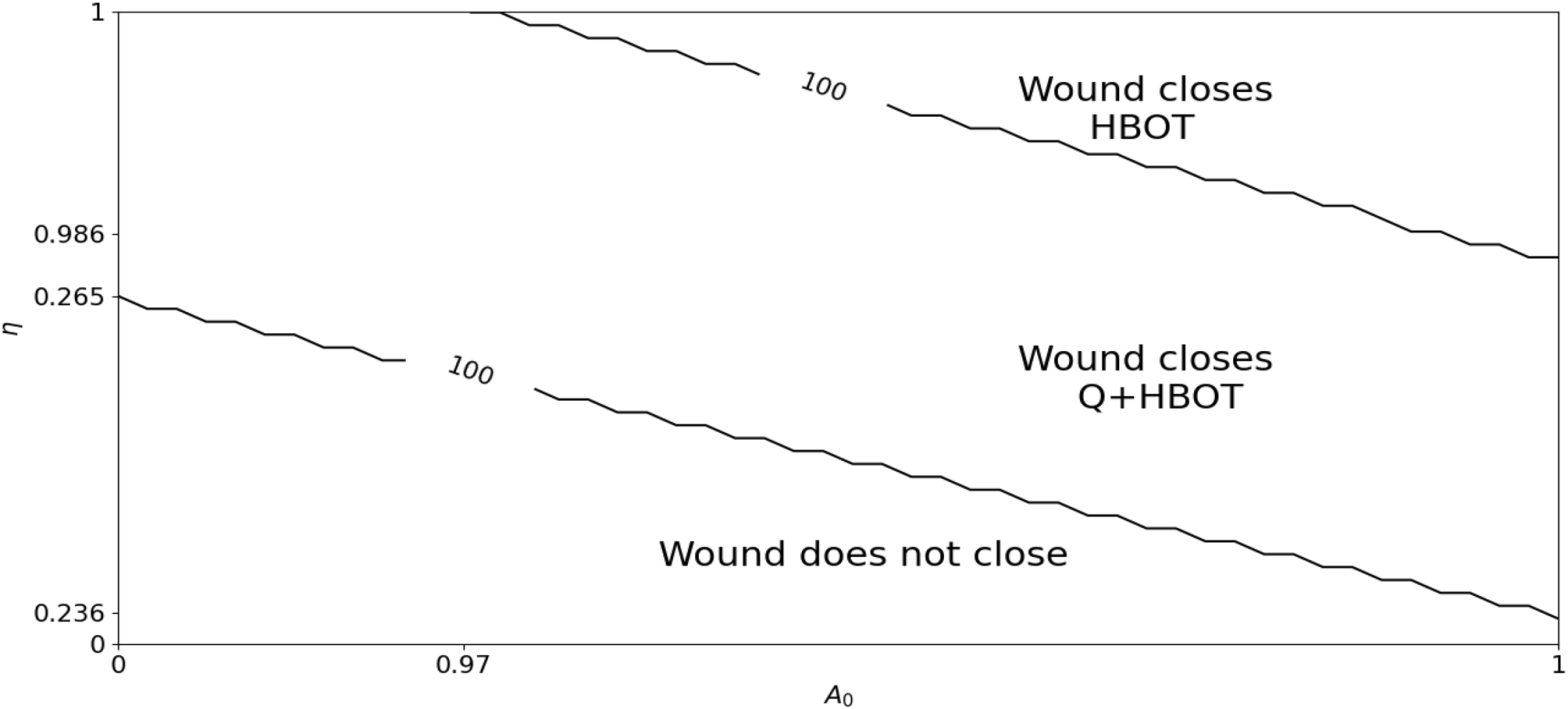
Effect of age $(\eta ,A_0)$ on percent wound closure: Simulations PWC(30) under HBOT and Q+HBOT as the pair $(\eta ,A_0)$ vary. The region between the two curves represents the ages $(\eta ,A_0)$ under which wound closure is achieved under treatment with Q+HBOT, but not under treatment with HBOT alone.

Figure 5A is a color map of PWC(30) under treatment with HBOT, and Fig. 5B is a color map under treatment with Q+HBOT, where on the horizontal axis, $A_0$ reflects how well the organism performs angiogenesis functions, and on the vertical axis, $\eta$ represents the proliferation of senescent cells. The range of ($\eta ,A_0$) was restricted in order to display, more clearly, in each of the figures, the region where PWC(30)$=100\%$.

Based on Fig. 5, Figure 6 shows the region in the $(\eta ,A_0)$ plane where treatment with HBOT does not achieve wound closure in 30 days, but treatment with Q+HBOT does. This region lies between two equi-PWC curves: The upper curve includes the extreme points (1, 0.97) and (0.986, 1), and the lower once includes the extreme points (0.265, 0) and (0.236, 1).

## Discussion

Diabetic wounds represent a growing global health crisis, with approximately 15-25% of diabetic patients developing chronic foot ulcers that frequently lead to infections and amputations^51^. Current treatment modalities include advanced wound dressings, negative pressure therapy, hyperbaric oxygen therapy (HBOT), and emerging biologic therapies, but these approaches often fail to address the underlying pathophysiology of impaired healing in diabetic patients^52^. The economic burden is staggering, with annual U.S. costs ranging between $9 to $13 billion due to prolonged hospitalizations, frequent debridements, and high recurrence rates; in addition to the cost for management of diabetes mellitus alone^53,54^. These costs are significantly lower in European systems like England’s NHS and France’s universal healthcare, where standardized treatment protocols, preventive care models, and price controls reduce both complication rates and expenditures^55,56^. The medical community in the U.S. has proposed several cost-reduction strategies, including value-based care models, early screening programs, and the adoption of senolytic therapies to target cellular senescence – a key contributor to impaired wound healing^57^. However, significant obstacles remain, including fragmented healthcare delivery, inconsistent insurance coverage, and a lack of reliable biomarkers to predict treatment response.

This paper contributes to addressing these challenges by developing a mathematical model that quantifies biological aging through two key parameters ($\eta$ for fibroblast proliferation and $A_0$ for VEGF production) to predict patient responsiveness to HBOT and senolytic therapy with quercetin (*Q*). Our simulations demonstrate that while HBOT alone is effective only for a limited subset of patients with specific $(\eta , A_0)$ values, combining HBOT with *Q* expands the treatable population by addressing cellular senescence^16^. This approach could reduce costs by enabling clinicians to: (1) avoid ineffective HBOT in non-responders, (2) identify patients who would benefit from adjunct senolytic therapy, and (3) potentially shorten treatment duration through targeted interventions. While the model requires clinical validation to correlate $\eta$ and $A_0$ with measurable biomarkers, it provides a quantitative framework for personalized treatment decisions that could optimize resource allocation in diabetic wound care^58^.

## Conclusion

Cellular senescence is a permanent arrest of cell cycle while maintaining viability. Cellular senescence is the hallmark of aging. Senescent cells secrete proteins that have negative effect on tissue regeneration. Diabetic wounds, wounds that develop in individuals with type 2 diabetes, tend to be ischemic due to the inflammation induced by the diabetic condition. And in aging individuals with diabetes type 2, due to also cellular senescence of fibroblasts, diabetic wounds are more likely to become chronic wounds.

Ischemic wounds are commonly treated with oxygen infusion, where the patient spends several hours per day in hyperbaric oxygen therapy chamber (HBOT) under oxygen pressure three times the pressure in air. Senolytic drugs that eliminate senescent cells, such as quercetin (*Q*), are currently used in experimental studies in chronic wounds. In this paper we simulate treatments with HBOT and *Q*, separately or in combination, of diabetic wounds in aging population.

To better understand the impact of aging on wound healing, we developed a predictive mathematical model that defines an individual’s biological age using two key parameters: $\eta$ and $A_0$. These parameters decrease with chronological age, where $0 \le \eta \le 1$ and $0 \le A_0 \le 1$. The parameter $\eta$ reflects age-related changes in the proliferation of senescent cells, while $A_0$ represents age-related production of the angiogenetic protein VEGF. Lower values of $\eta$ and $A_0$ indicate a biologically older individual. Our model can be used to predict the profile of the open-wound radius, with or without treatment, for any biological-age pair $(\eta ,A_0)$.

We simulated the threshold of two $(\eta ,A_0)$-sets, $S_1$ and $S_2$ ($S_1\subset S_2$): patients in $S_1$ achieve wound closure (in expectable time of 30 days) if treated with HBOT, and patients in $S_2$ achieve ful wound closure when treated with *Q*+HBOT. We conclude that for the biologically-old patients in $S_2-S_1$, to achieve wound closure they need to be treated with combination of the senolytic drug and HBOT; HBOT alone will not yield wound closure.

The limitation of the model is that it is unable to precisely associate the biological-age parameters $(\eta ,A_0)$ to any tangible marks that can be determined and measured for each individual. For this reason, the predictions of the model, at present, are highly qualitative.

Future studies should aim to determine the dependence of $\eta$ and $A_0$ on factors such as lifestyle, general health, and body mass to further personalize treatment strategies. This would significantly improve the predictive accuracy of our model and allow for more individualized care. Ultimately, the integration of these parameters into clinical practice could significantly improve the management of chronic wounds in the aging diabetic population, providing clinicians with a tool to better predict healing outcomes and optimize treatment plans.

## Parameters sensitivity analysis

**Fig. 7:**
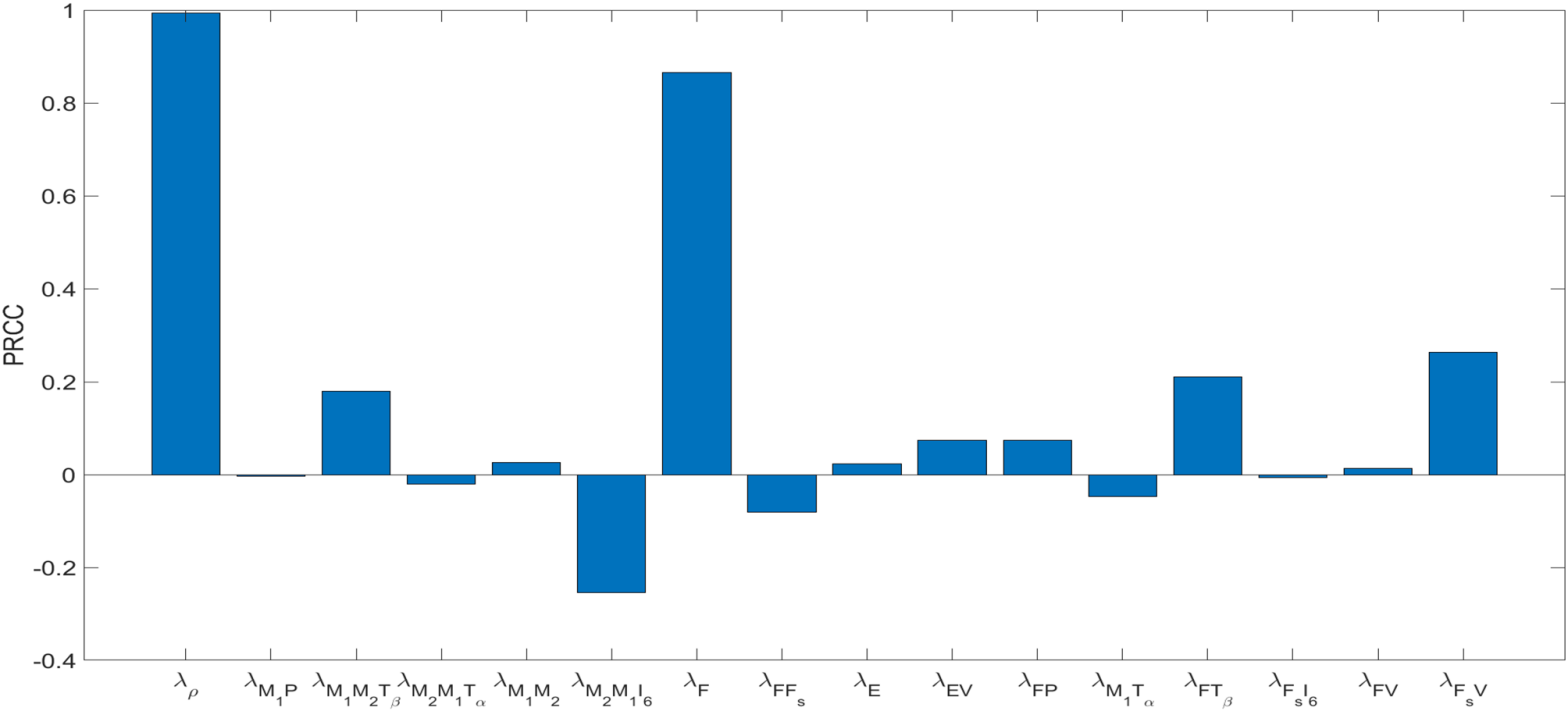
LHS–PRCC sensitivity analysis of radius reduction at day 30. The horizontal axis lists the parameters and the vertical axis represents the PRCC index. The samplings are done with $\pm 50\%$ of the baselines for the parameters as in Tables 4, except for $\lambda _\rho$ whose range is $\pm 10\%$ of the baseline.

We performed global sensitivity analyses (see Fig. 7). The output was the percent wound closure at day 30 (PWC), and the p-values for all the parameters were less than $10^{-8}$. The computations were done using Latin Hypercube Sampling/Partial Rank Correlation Coefficient (LHS/PRCC) with a Matlab package by^59,60^, with the parameters listed $\lambda _\rho$, $\lambda _{M_1P}$, $\lambda _{M_1M_2T_\beta }$, $\lambda _{M_2M_1T_\alpha }$, $\lambda _{M_1M_2}$, $\lambda _{M_2M_1I_6}$, $\lambda _F$, $\lambda _{FF_s}$, $\lambda _E$, $\lambda _{EV}$, $\lambda _{FP}$, $\lambda _{M_1T_\alpha }$, $\lambda _{FT_\beta }$, $\lambda _{F_sI_6}$, $\lambda _{FV}$ and $\lambda _{F_sV}$ in Table 4.

The ranges for the parameters were between $\pm 50\%$ of their baselines in Table 4, except $\lambda _\rho$ whose range was between $\pm 10\%$ of its baseline in Table 4.

Figure 7 shows that $\lambda _\rho$ and $\lambda _F$ are the most positive correlated parameters, with $\lambda _{M_1M_2T_\beta }$, $\lambda _{FT_\beta }$ and $\lambda _{F_sV}$ also showing significant positive correlations. The parameter $\lambda _\rho$ represents the rate of ECM production; as it increases, ECM production rises, leading to a reduction in wound size and an increase in PWC. Similarly, an increase in $\lambda _F$, which denotes the rate of fibroblast proliferation, results in enhanced ECM production and a larger PWC. Additionally, increases in the parameters $\lambda _{M_1M_2T_\beta }$ and $\lambda _{FT_\beta }$ promote the $M_1 \rightarrow M_2$ polarization, further contributing to increased ECM production and larger PWC. An increase in $\lambda _{F_sV}$ leads to an increase in *V*, which in turn results in an increase in *E*. This implies that *w* also increases, thereby enhancing the wound healing process. Note that the positive correlation of $\lambda _{FV}$ is smaller than the positive correlation of $\lambda _{F_sV}$; this is because $\lambda _{FV}$ is 10 times smaller than $\lambda _{F_sV}$.

The reverse is true for the parameters $\lambda _{M_2M_1I_6}$, $\lambda _{M_2M_1T_\alpha }$ and $\lambda _{M_1T_\alpha }$, which are the most negatively correlated parameters. As $\lambda _{M_2M_1I_6}$ and $\lambda _{M_2M_1T_\alpha }$ increase, the density of M1 macrophages also increases, which decreases PWC. An increase in $\lambda _{M_1T_\alpha }$ leads to higher levels of TNF-$\alpha$, which similarly results in an increase in M1 macrophages, and decrease PWC.

All other parameters have small correlations, and their positive or negative correlation can easily be inferred from the model equations. For instance, increase in any parameter that results in increase $M_1\rightarrow M_2$ will improve healing and hence increase PWC, and is positively correlated.

## Parameter estimations

We assume that in steady-state, $\frac{X}{K_X+X}=\frac{1}{2}$, or $X=K_X$ for any protein species, *X*, where $K_X$ is the half-saturation of *X*.

### Densities/concentrations in steady state

**Estimate for** $\rho _0,\ \rho _m,\ \rho _1$ **and** $\gamma$**.** The ECM density is 3–4% of the dry weight of tissue^61^; we accordingly take

$$\begin{aligned}\rho _0=0.04\ \text {g/cm}^3.\end{aligned}$$

We also take

$$\begin{aligned}\rho _m=1.1\rho _0=0.044\ \text {g/cm}^3,\ \rho _1=0.2\rho _0=0.008\ \text {g/cm}^3,\end{aligned}$$

and

$$\begin{aligned}\gamma =0.19\ \text {d}^{-1}.\end{aligned}$$

#### Estimate for $w_0$.

The concentration of oxygen in tissue is given by the following formula (in text, section Materials and Methods of^62^):

$$\begin{aligned}w_0=P_{O_2}\times \alpha _{tissue},\end{aligned}$$

where $P_{O_2}=100$ M/mmHg is the oxygen pressure in arterial blood and (from Table 3 in^62^) $\alpha _{tissue}=1.25\times 10^{-6}\ \text {mmHg}$ is the oxygen solubility in the tissue ($\text {M}=10^{-3}\frac{\text {mol}}{\text {cm}^3}=32\times 10^{-3}\frac{\text {g}}{\text {cm}^3}$). Hence

$$\begin{aligned}w_0=125\times 10^{-6}\ \text {M}=4\times 10^{-6}\ \text {g/cm}^3.\end{aligned}$$

*F* and $M_2$ cells undergo increased apoptosis as the oxygen concentration (in g/$\hbox {cm}^3$) decreases from $4\times 10^{-6}$ to $3.2\times 10^{-7}$. We assume that the rate of apoptosis is porportional to *D*(*w*) and take

$$\begin{aligned}w_i=2.5\times 10^{-6}\ \text {g/cm}^3.\end{aligned}$$

#### Estimates for steady $M_1^0$ (of $M_1$) and $M_2^0$ (of $M_2$).

There are 2–4$\times 10^4$ macrophages per $\hbox {mm}^3$ in mid-dermis^63^. We take the number of macrophages to be $3\times 10^4$ cells/$\hbox {mm}^3$ and the mass of a cell to be $5\times 10^{-10}$ g. We assume that $M_1^0>M_2^0$ in the steady state of mid-epidermis, and take

$$\begin{aligned}M_1^0=2.5\times 10^{-3}\ \text {g/cm}^3,\end{aligned}$$

and

$$\begin{aligned}M_2^0=5M_1^0=1.25\times 10^{-2}\ \text {g/cm}^3.\end{aligned}$$

#### Estimate for $F_0$.

There are 2100–4100 fibroblasts/$\hbox {mm}^3$ in mid-dermis^64^. The volume of a fibroblast cell is $2.5\times 10^{-9}$ $\hbox {cm}^3$^65^(Fig. 1B) and accordingly we take its mass to be $2.5\times 10^{-9}$ g. Assuming that there are 3000 fibroblasts cells in $\hbox {mm}^3$, we get the steady-state of *F* to be

$$\begin{aligned}F_0=3\times 10^6\times 2.5\times 10^{-9}=7.5\times 10^{-3}\ \text {g/cm}^3.\end{aligned}$$

#### Estimate for $F_s^0$

Wounds are hard to heal when the accumulation of senescent fibroblasts exceeds 15% threshold^66^. For diabetic wounds we take

$$\begin{aligned}F_s^0=40\%F_0=3\times 10^{-3}\ \text {g/cm}^3.\end{aligned}$$

#### Estimate for $E_0$.

The number of human corneal endothelial cells is 3000 cells/$\hbox {mm}^2$, or $3\times 10^5$ cells/$\hbox {cm}^2$^67,68^. We assume that the number of endothelial cells in mid-dermis is $2\times 10^7$ cells/$\hbox {cm}^3$. Taking the mass of one cell to be $5\times 10^{-10}$ g, we get the steady-state of *E*:

$$\begin{aligned}E_0=2\times 10^7\times 5\times 10^{-10}=10^{-2}\ \text {g/cm}^3.\end{aligned}$$

#### Estimate for $K_{I_6}$

The level of IL-6 in normal human skin is approximately 205.1 pg/g^69^(Tab. 1).

The level of IL-6 in human skin wounds is increasing after wound initiation^69^(Fig. 4). We take the average level of IL-6 around the wound to be

$$\begin{aligned}K_{I_6}=205.1\times 2=4.102\times 10^{-10}\ \text {g/cm}^3.\end{aligned}$$

#### Estimate for $K_{T_\beta }$

In humans, the level of TGF-$\beta$ decreases from its high value within the first hours post wound initiation to reach an average of 100 pg/$\hbox {mm}^3$^70^(Fig. 2).

Since diabetic wounds do not heal without intervention, we take

$$\begin{aligned}K_{T_\beta }=100\ \text {pg/mm}^3=10^{-7}\ \text {g/cm}^3.\end{aligned}$$

#### Estimate for $K_P$

In mice, the level of PDGF in the wound was measured to be approximately 6 ng/mg at day 3 post wound initiation and 1.5 ng/mg at day 7 post wound initiation^71^(Fig. 2c).

We take in chronic wounds,

$$\begin{aligned}K_P=4\ \text {ng/mg}=4\times 10^{-6}\ \text {g/cm}^3.\end{aligned}$$

#### Estimate for $K_V$

We assume that the average level of VEGF after wound initiation is similar to that of murine skin 5 to 7 days after wound initiation^9^(Fig. 7) and take

$$\begin{aligned}K_V=1150\ \text {pg/mg}=1.15\times 10^{-6}\ \text {g/cm}^3.\end{aligned}$$

#### Estimate for $K_{T_\alpha }$

In murine skin, the level of TNF-$\alpha$ increases from its control level (60 pg/ml) to 120 pg/ml after wound initiation^72^(Figs. 1,2). We take, in diabetic wounds,

$$\begin{aligned}K_{T_\alpha }=100\ \text {pg/ml}=10^{-10}\ \text {g/cm}^3.\end{aligned}$$

#### Death/degradation rates

The death or degradation rate $\mu _X$ of a species *X* is linked to its half-life $t_{1/2}(X)$ by the formula:

$$\begin{aligned}\mu _X=\dfrac{\ln 2}{t_{1/2}(X)}.\end{aligned}$$

**Estimate for** $\mu _{I_6}$**.** The half-life of IL-6 is 15.5 hours^73^, or equivalently 0.646 days. Hence,

$$\begin{aligned}\mu _{I_6}=1.073\ \text {d}^{-1}.\end{aligned}$$

**Estimate for** $\mu _P$**.** The half-life of PDGF is 30 minutes^74^. We take $t_{1/2}(P)=0.021$ days, so that

$$\begin{aligned}\mu _P=33\ \text {d}^{-1}.\end{aligned}$$

**Estimate for** $\mu _V$**.** The half-life of VEGF is 60 minutes^75^. Hence $t_{1/2}(V)=0.042$ days, and

$$\begin{aligned}\mu _V=16.5\ \text {d}^{-1}.\end{aligned}$$

**Estimate for** $\mu _{T_\alpha }$**.** The half-life of TNF-$\alpha$ is 4.6 minutes^76^. Hence $t_{1/2}(T_\alpha )=3.2\times 10^{-3}$ days, and

$$\begin{aligned}\mu _{T_\alpha }=216.61\ \text {d}^{-1}.\end{aligned}$$

**Estimate for** $\mu _{T_\beta }$**.** The half-life of TGF-$\beta$ is approximately 2 minutes^77^. Hence $t_{1/2}(T_\beta )=1.4\times 10^{-3}$ days, and

$$\begin{aligned}\mu _{T_\beta }=495.1\ \text {d}^{-1}.\end{aligned}$$

**Estimate for** $\mu _{M_1}$**.** The half-life of tissue M1 macrophages is three weeks^78^, or, equivalently, 21 days. Hence,

$$\begin{aligned}\mu _{M_1}=0.033\ \text {d}^{-1}.\end{aligned}$$

**Estimate for** $\mu _{M_2}$**.** The half-life of tissue M2 macrophages is 7 days^78^, so that

$$\begin{aligned}\mu _{M_2}=0.099\ \text {d}^{-1}.\end{aligned}$$

**Estimate for** $\mu _F$ **and** $\mu _{F_s}$**.** The death rate of fibroblast is approximately $2.31\times 10^{-7}$ per second^79^, hence,

$$\begin{aligned}\mu _F=2.31\times 10^{-7}\times 24\times 60\times 60=0.02\ \text {d}^{-1}.\end{aligned}$$

We take

$$\begin{aligned}\mu _{F_s}=1.25\mu _F=0.025\ \text {d}^{-1}.\end{aligned}$$

**Estimate for** $\mu _E$**.** The half-life of cardiac endothelial cells is 2.2 weeks^80^, or, equivalently, 15.4 days. Using the same half-life for dermal epithelial cells, we get

$$\begin{aligned}\mu _E=0.045\ \text {d}^{-1}.\end{aligned}$$

#### Chemotactic coefficients

We take

$$\begin{aligned}\chi _P=\chi _V=1000\ \text {cm}^5/\text {g}\cdot \text {d},\end{aligned}$$

which is in the range given in^81^ for $\chi _P$.

#### Estimates by equations

There are still many parameters that need to be estimated, including all the production coefficients. To do that we shall take, in each equation, its steady state, that is, make the right-hand side equal to zero, drop chemotactic terms, and replace each species by its steady state as estimated above.

- Equation (3): From the steady state equation (3) in healthy tissue (i.e., with $T_\beta =0$), we get
  $$\begin{aligned}\lambda _\rho F(1-\rho _0/\rho _m)=\mu _\rho \rho _0,\end{aligned}$$
  where $F=F_0=7.5\times 10^{-3}$ g/$\hbox {cm}^3$, $\rho _m=1.1\rho _0$, $\mu _\rho =0.37$ $\hbox {d}^{-1}$ by^36^, and $\rho _0=0.04$ g/$\hbox {cm}^3$. Hence
  $$\begin{aligned}\lambda _\rho =21.71 \text {d}^{-1}.\end{aligned}$$
  We take
  $$\begin{aligned}\lambda _{\rho T_\beta }=0.1.\end{aligned}$$
- Equations (4) and (5): From the steady state equation (4) in healthy state, we get $A_{M_1}=\mu _{M_1}M_1^0$, where $\mu _{M_1}=0.033$ $\hbox {d}^{-1}$ and $M_1^0=2.5\times 10^{-3}$ g/$\hbox {cm}^3$. Hence,
  $$\begin{aligned}A_{M_1}=7.5\times 10^{-5}\ \text {g/cm}^3\, \text {d}^{-1}.\end{aligned}$$
  We take $\lambda _{M_1P}=0.2\mu _{M_1}=6.6\times 10^{-3}$ $\hbox {d}^{-1}$ and $\lambda _{M_1M_2}=\lambda _{M_1P}/2=3.3\times 10^{-4}$ $\hbox {d}^{-1}$. We assume that $\lambda _{M_2M_1I_6}>\lambda _{M_1M_2T_\beta }>\lambda _{M_2M_1T_\alpha }$ and take $\lambda _{M_2M_1I_6}=2\lambda _{M_1M_2T_\beta }$ and $\lambda _{M_1M_2T_\beta }=11\lambda _{M_2M_1T_\alpha }$. Then from the steady state of Eq. (4)
  $$\begin{aligned}A_{M_1}+\lambda _{M_1P}M_1^0/2-11\lambda _{M_2M_1T_\alpha }M_1^0/2+\lambda _{M_2M_1T_\alpha }M_2^0/2-\lambda _{M_1M_2}M_1^0+\lambda _{M_2M_1T_\alpha }M_2^0/2-\mu _{M_1}M_1^0=0\end{aligned}$$
  where $M_1^0=2.5\times 10^{-3}$ g/$\hbox {cm}^3$ and $M_2^0=1.25\times 10^{-2}$ g/$\hbox {cm}^3$, we get
  $$\begin{aligned}\lambda _{M_1M_2T_\beta }=5.94\times 10^{-3}\ \text {d}^{-1}, \lambda _{M_2M_1T_\alpha }=6.53\times 10^{-2}\ \text {d}^{-1}\text { and } \lambda _{M_2M_1I_6}=5.94\times 10^{-3}\ \text {d}^{-1}.\end{aligned}$$
  All the coefficients in Eq. (5) are now estimated except $\mu _{M_2w}$. We take
  $$\begin{aligned}\mu _{M_2w}=\mu _{M_2}/10=9.9\times 10^{-3}\ \text {d}^{-1}.\end{aligned}$$
- Equation (6): We take $F_m=2F_0=15\times 10^{-3}$ g/$\hbox {cm}^3$ and $\lambda _{FF_s}=\lambda _F/10$. In steady state of health, $\lambda _FF(1-F/F_m)/2-\lambda _FF/10-\mu _FF=0$. Since $\mu _F=0.02$ $\hbox {d}^{-1}$, we get
  $$\begin{aligned}\lambda _F=0.15\ \text {d}^{-1}\text { and }\lambda _{FF_s}=0.015\ \text {d}^{-1}.\end{aligned}$$
  We take
  $$\begin{aligned}\mu _{Fw}=\mu _F/10=2\times 10^{-3}\ \text {d}^{-1}.\end{aligned}$$
- Equation (8): We take $\lambda _{EV}=1$. Then the steady state reduces to $3\lambda _E/2-\mu _EE_0=0$, where $\mu _E=0.045$ $\hbox {d}^{-1}$ and $E_0=10^{-2}$ g/$\hbox {cm}^3$, so that
  $$\begin{aligned}\lambda _E=3\times 10^{-4}\ \text {g/cm}^3\,\text {d}^{-1}.\end{aligned}$$
- Equation (9): We take the “average” value of *R*(*t*) in diabetic wounds control case to be ${\bar{R}}=0.7$ cm, and assume that $\lambda _P{\bar{R}}-\mu _PK_P=0$, so that
  $$\begin{aligned}\lambda _P=1.19\times 10^{-4}\ \text {g/cm}^3\, \text {d}^{-1}.\end{aligned}$$
  Next, in steady-state, $\lambda _{FP}F=(\mu _{PF}F+\mu _{PM}M_1)/2$. We assume that $\mu _{PF}=\mu _{PM}=\theta \lambda _P$ for some $\theta >0$. Taking $\theta =1.23$, we get $\lambda _P+\lambda _{FP}F-\mu _{PF}(F+M_1)/2-\mu _PK_P=0$. Hence,
  $$\begin{aligned}\mu _{PF}=\mu _{PM}=1.043\times 10^{-4}\ \text {d}^{-1},\ \text {and}\ \lambda _{FP}=7\times 10^{-5}\ \text {d}^{-1}.\end{aligned}$$
- Equation (10): The steady state equation is $\lambda _{M_1T_\alpha }M_1^0-\mu _{T_\alpha }K_{T_\alpha }=0$, where $M_1^0=2.5\times 10^{-3}$ g/$\hbox {cm}^3$, $K_{T_\alpha }=10^{-10}$ g/$\hbox {cm}^3$ and $\mu _{T_\alpha }=216.6$ $\hbox {d}^{-1}$. Hence,
  $$\begin{aligned}\lambda _{M_1T_\alpha }=8.664\times 10^{-6}\ \text {d}^{-1}.\end{aligned}$$
- Equation (11): We take $\lambda _{FT_\beta }=\lambda _{M_2T_\beta }$. The steady state equation then becomes $\lambda _{FT_\beta }(M_2^0+F_0)-\mu _{T_\beta }K_{T_\beta }=0$, where $M_2^0=1.25\times 10^{-2}$ g/$\hbox {cm}^3$, $F_0=7.5\times 10^{-3}$ g/$\hbox {cm}^3$, $K_{T_\beta }=10^{-7}$ g/$\hbox {cm}^3$, and $\mu _{T_\beta }=495.1$ $\hbox {d}^{-1}$. Hence,
  $$\begin{aligned}\lambda _{FT_\beta }=\lambda _{M_2T_\beta }=2.5\times 10^{-3}\ \text {d}^{-1}.\end{aligned}$$
- Equation (12): The steady-state is given by $\lambda _{F_sI_6}F_s^0-\mu _{I_6}K_{I_6}=0$, where $F_s^0=3\times 10^{-3}$ g/$\hbox {cm}^{-3}$, $K_{I_6}=4.102\times 10^{-10}$ g/$\hbox {cm}^{-3}$, and $\mu _{I_6}=1.073$ $\hbox {d}^{-3}$. Hence,
  $$\begin{aligned}\lambda _{F_sI_6}=2.9343\times 10^{-7}\ \text {d}^{-1}.\end{aligned}$$
- Equation (13): $F_s$ secretes significantly more VEGF than *F*^40,41^. We take $\lambda _{F_sV}=10\lambda _{FV},\ \lambda _{FV}=\lambda _{M_2V},\ \mu _{EV}=1.2\mu _V$, and write the time-average steady-state with the average value of *A*(*t*) over 30 days, ${\bar{A}}=\frac{1}{30}\int _0^{30}\frac{A_0}{1+t^2/T_w^2}$ with $A_0=0.5$ and $T_w=50$ d, as follows: ($\lambda _{FV}F_0+10\lambda _{FV}F_s^0){\bar{A}}+\lambda _{FV}M_2^0-1.2\mu _VE_0/2-\mu _VK_V=0$, where $M_2^0=1.25\times 10^{-2}$ g/$\hbox {cm}^{-3}$, $E_0=10^{-2}$ g/$\hbox {cm}^{-3}$, $K_V=1.15\times 10^{-6}$ g/$\hbox {cm}^3$, $\mu _V=16.5$ $\hbox {d}^{-1}$ and ${\bar{A}}=0.45035$. Hence,
  $$\begin{aligned}\lambda _{FV}=3.17\ \text {d}^{-1},\ \lambda _{F_sV}=31.7\ \text {d}^{-1},\ \lambda _{M_2V}=3.17\ \text {d}^{-1}\ \text {and}\ \mu _{EV}=19.8\ \text {d}^{-1}.\end{aligned}$$
- Equation (15): From the steady state in health, $\lambda _{Ew} E_0-c_w(F_0+M_2^0)w_0=0$, where $E_0=10^{-2}$ g/$\hbox {cm}^{-3}$, $F_0=7.5\times 10^{-3}$ g/$\hbox {cm}^3$, $M_2^0=1.25\times 10^{-2}$ g/$\hbox {cm}^3$ and $w_0=4\times 10^{-6}$ g/$\hbox {cm}^3$. Hence,
  $$\begin{aligned}\lambda _{Ew}=8\times 10^{-6}\times c_w\ \text {d}^{-1}.\end{aligned}$$
  Taking
  $$\begin{aligned}c_w=0.3\ \text {cm}^3/\text {g d}^{-1},\ \text {we get}\ \lambda _{Ew}=2.4\times 10^{-6}\ \text {d}^{-1}.\end{aligned}$$

**Table 4:** Parameters for the model.

Parameters	Descriptions	Values	References
$\lambda _\rho$	Rate of production of ECM by *F*	21.71 $\hbox {d}^{-1}$	est.
$\lambda _{M_1P}$	Rate of recruitment of $M_1$	$6.6\times 10^{-3}$ $\hbox {d}^{-1}$	est.
$\lambda _{M_1M_2T_\beta }$	Rate of $T_\beta$-induced transition $M_1\rightarrow M_2$	$6.53\times 10^{-2}$ $\hbox {d}^{-1}$	est.
$\lambda _{M_2M_1T_\alpha }$	Rate of $T_\alpha$-induced transition $M_2\rightarrow M_1$	$5.94\times 10^{-3}$ $\hbox {d}^{-1}$	est.
$\lambda _{M_2M_1I_6}$	Rate of $I_6$-induced transition $M_2\rightarrow M_1$	$5.94\times 10^{-3}$ $\hbox {d}^{-1}$	est.
$\lambda _{M_1M_2}$	Rate of transition $M_1\rightarrow M_2$	$3.3\times 10^{-4}$ $\hbox {d}^{-1}$	est.
$\lambda _F$	Rate of proliferation of *F*	0.15 $\hbox {d}^{-1}$	est.
$\lambda _{FF_s}$	Rate of transition $F\rightarrow F_s$	0.015 $\hbox {d}^{-1}$	est.
$\lambda _E$	Rate of maturation of *E* by EPC	$3\times 10^{-4}$ $\hbox {d}^{-1}$	est.
$\lambda _P$	Recruitment rate of *P*	$1.19\times 10^{-4}$ g/$\hbox {cm}^2$ $\hbox {d}^{-1}$	est.
$\lambda _{FP}$	Rate of production of *P* by *F*	$7\times 10^{-5}$ $\hbox {d}^{-1}$	est.
$\lambda _{M_1T_\alpha }$	Rate of production of $T_\alpha$	$8.664\times 10^{-6}$ $\hbox {d}^{-1}$	est.
$\lambda _{FT_\beta }$	Rate of production of $T_\beta$ by *F*	$2.5\times 10^{-3}$ $\hbox {d}^{-1}$	est.
$\lambda _{M_2T_\beta }$	Rate of production of $T_\beta$ by $M_2$	$2.5\times 10^{-3}$ $\hbox {d}^{-1}$	est.
$\lambda _{\rho T_\beta }$	$T_\beta$-factor of $\rho$-production by *F*	0.1	est.
$\lambda _{M_2V}$	Rate of production of *V* by $M_2$	3.17 $\hbox {d}^{-1}$	est.
$\lambda _{FV}$	Rate of production of *V* by *F*	3.17 $\hbox {d}^{-1}$	^40,41^est.
$\lambda _{F_sV}$	Rate of production of *V* by $F_s$	31.7 $\hbox {d}^{-1}$	^40,41^est.
$\lambda _{F_sI_6}$	Rate of production of $I_6$ by $F_s$	$2.9343\times 10^{-7}$ $\hbox {d}^{-1}$	^66^est.
$\lambda _{Ew}$	Rate of circulation of oxygen	$2.4\times 10^{-6}$ $\hbox {d}^{-1}$	est.
$\lambda _{FQ}$	Rate of Q-enhanced proliferation of *F*	0.2 d $\hbox {g}^{-1}$	est.
$c_w$	Rate of consumption of oxygen	0.3 $\hbox {cm}^3$/g $\hbox {d}^{-1}$	est.
$\gamma$	Factor of internal isotropic medium	0.19 $\hbox {d}^{-1}$	est.
$\chi _P$	Chemotactic coefficient of *P*	1000 $\hbox {cm}^5$/g$\cdot$d	^81^est.
$\chi _V$	Chemotactic coefficient of *V*	1000 $\hbox {cm}^5$/g$\cdot$d	^81^est.
$\lambda _{\rho T_\beta }$	Fraction of effect of $T_\beta$ on production of $\rho$ by *F*	0.1	est.
$\lambda _{EV}$	Coefficient of maturation of *E* by *V*	1	est.
$K_{T_\beta }$	Half-saturation of $T_\beta$	$10^{-7}$ g/$\hbox {cm}^3$	^70^est.
$K_{I_6}$	Half-saturation of $I_6$	$4.102\times 10^{-10}$ g/$\hbox {cm}^3$	^69^est.
$K_P$	Half-saturation of *P*	$4\times 10^{-6}$ g/$\hbox {cm}^3$	^71^est.
$K_{T_\alpha }$	Half-saturation of $T_\alpha$	$10^{-10}$ g/$\hbox {cm}^3$	^72^est.
$K_V$	Half-saturation of *V*	$1.15\times 10^{-6}$ g/$\hbox {cm}^3$	^9^est.
$\rho _0$	Healthy state of ECM	0.04 g/$\hbox {cm}^3$	^61^est.
$\rho _1$	Threshold of pressure of ECM	0.008 g/$\hbox {cm}^3$	^61^est.
$\rho _m$	Carrying capacity of ECM	0.044 g/$\hbox {cm}^3$	^61^est.
$A_{M_1}$	Recruitment rate of $M_1$	$7.5\times 10^{-5}$ g/$\hbox {cm}^3$ $\hbox {d}^{-1}$	est.
$w_0$	Oxygen concentration	$4\times 10^{-6}$ g/$\hbox {cm}^3$	^82,83^est.
$w_i$	Oxygen concentration in extreme hypoxia	$2.5\times 10^{-6}$ g/$\hbox {cm}^3$	^82,83^est.
$M_1^0$	Steady state in health of $M_1$	$2.5\times 10^{-3}$ g/$\hbox {cm}^3$	^63^est.
$M_2^0$	Steady state in health of $M_1$	$1.25\times 10^{-2}$ g/$\hbox {cm}^3$	^63^est.
$F_0$	Steady state in health of *F*	$7.5\times 10^{-3}$ g/$\hbox {cm}^3$	^64,65^est.
$F_s^0$	Steady state in health of $F_s$	$1.5\times 10^{-3}$ g/$\hbox {cm}^3$	^66^est.
$E_0$	Steady state in health of *E*	$10^{-2}$ g/$\hbox {cm}^3$	^67,68^est.
$\mu _\rho$	Degradation rate of ECM	0.37 $\hbox {d}^{-1}$	^36^
$\mu _P$	Degradation rate of PDGF	33 $\hbox {d}^{-1}$	^84^est.
$\mu _{T_\alpha }$	Degradation rate of TNF-$\alpha$	216.6 $\hbox {d}^{-1}$	^76^est.
$\mu _{T_\beta }$	Degradation rate of TGF-$\beta$	495.1 $\hbox {d}^{-1}$	^85^est.
$\mu _V$	Degradation rate of VEGF	16.5 $\hbox {d}^{-1}$	^86^est.
$\mu _w$	Degradation rate of oxygen	$8.33\times 10^{-3}$ $\hbox {d}^{-1}$	^87^est.
$\mu _{M_1}$	Death rate of $M_1$	0.033 $\hbox {d}^{-1}$	^78^est.
$\mu _{M_2}$	Death rate of $M_2$	0.099 $\hbox {d}^{-1}$	^78^est.
$\mu _E$	Death rate of endothelial cells	0.045 $\hbox {d}^{-1}$	^80^est.
$\mu _F$	Death rate of *F*	0.02 $\hbox {d}^{-1}$	^88^est.
$\mu _{F_s}$	Death rate of $F_s$	0.025 $\hbox {d}^{-1}$	est.
$\mu _{I_6}$	Death rate of $I_6$	1.073 $\hbox {d}^{-1}$	^73^est.
$\mu _{PF}$	Depletion rate of *P* by *F*	$1.043\times 10^{-4}$ $\hbox {d}^{-1}$	est.
$\mu _{PM}$	Depletion rate of *P* by *M*	$1.043\times 10^{-4}$ $\hbox {d}^{-1}$	est.
$\mu _{EV}$	Depletion rate of *E* by *V*	19.8 $\hbox {d}^{-1}$	est.
$\mu _{M_2w}$	Depletion rate of $M_2$ by *w*	$9.9\times 10^{-3}$ $\hbox {d}^{-1}$	est.
$\mu _{Fw}$	Death rate of *F* by *w*	$2\times 10^{-3}$ $\hbox {d}^{-1}$	est.
$\mu _{\rho Q}$	Rate of Q-enhanced secretion of $\rho$ by *F*	$5\times 10^{-3}$ d $\hbox {g}^{-1}$	est.
$\mu _{F_sQ}$	Rate of elimination of $F_s$ by Q	$5\times 10^{-2}$ $\hbox {g}^{-1}$	est.
$D_{M_1}$	Diffusion coefficient of $M_1$	$8.64\times 10^{-7}$ $\hbox {cm}^2\hbox {d}^{-1}$	^89^est.
$D_{M_2}$	Diffusion coefficient of $M_2$	$8.64\times 10^{-7}$ $\hbox {cm}^2\hbox {d}^{-1}$	^89^est.
$D_E$	Diffusion coefficient of *E*	$8.64\times 10^{-7}$ $\hbox {cm}^2\hbox {d}^{-1}$	^89^est.
$D_F$	Diffusion coefficient of *F*	$8.64\times 10^{-7}$ $\hbox {cm}^2\hbox {d}^{-1}$	^89^est.
$D_{F_s}$	Diffusion coefficient of $F_s$	$8.64\times 10^{-7}$ $\hbox {cm}^2\hbox {d}^{-1}$	^89^est.
$D_P$	Diffusion coefficient of *P*	$8.63\times 10^{-2}$ $\hbox {cm}^2\hbox {d}^{-1}$	^90^est.
$D_{T_\alpha }$	Diffusion coefficient of $T_\alpha$	$8.48\times 10^{-2}$ $\hbox {cm}^2\hbox {d}^{-1}$	^91^est.
$D_{T_\beta }$	Diffusion coefficient of $T_\beta$	$7.1\times 10^{-2}$ $\hbox {cm}^2\hbox {d}^{-1}$	^91^est.
$D_V$	Diffusion coefficient of *V*	$8.64\times 10^{-2}$ $\hbox {cm}^2\hbox {d}^{-1}$	^92^est.
$D_w$	Diffusion coefficient of *w*	2 $\hbox {cm}^2\hbox {d}^{-1}$	^93^est.
$B_1$	Diabetic effect of oxygen flow blockade	10	This work
$B_2$	Diabetic effect of $M_1\rightarrow M_2$ blockade	2	This work
$T_w$	Diabetic effect of $M_1\rightarrow M_2$ blockade	50 d	This work

## Data Availability

All data generated or analyzed during this study are included in this published article.
